# Rapid Evolutionary Adaptation in Response to Selection on Quantitative Traits

**DOI:** 10.3390/life11080797

**Published:** 2021-08-06

**Authors:** Wolfgang Stephan

**Affiliations:** 1 Natural History Museum, 10115 Berlin, Germany; stephan@bio.lmu.de; 2 Faculty of Biology, Evolutionary Biology, Ludwig-Maximilian University of Munich, 82152 Planegg-Martinsried, Germany

**Keywords:** rapid adaptation, polygenic selection, quantitative traits

## Abstract

Evolutionary adaptation after sudden environmental changes can occur very rapidly. The mechanisms facilitating rapid adaptation range from strong positive directional selection leading to large shifts in the allele frequencies at a few loci (selective sweeps) to polygenic selection causing small changes in allele frequencies at many loci. In addition, combinations of these two extreme mechanisms may also result in fast evolution. In recent years, following reports of new case studies of rapid adaptation, population genetic models have been proposed to explain these observations. In these models, the role of the major selective forces (positive directional and stabilizing selection) is highlighted as well as the genetic architecture of quantitative traits. Furthermore, the factors limiting the speed of adaptation are analyzed, in particular, the effects of random genetic drift and demography due to finite population size.

## 1. Introduction 

Evolutionary adaptation can occur very rapidly. Well-known new case studies of rapid adaptation in response to environmental changes include those of the color variation in guppies [1], field mice [2] and peppered moth [3]; insecticide resistance in Drosophila [4]; beak size changes in Darwin’s finches [5], and limb development in Anolis lizards [6]. Yet, the genetic and evolutionary mechanisms causing fast adaptation are not well understood.

The genetic basis of these phenotypic traits spans a wide range, from only a few genes of major effect, such as in the peppered moth [7], to a large number of genes of very small effects at individual loci, such as in the case of human height [8]. Mirroring this wide spectrum of genetic architectures, the evolutionary genetic models that were proposed describe adaptation at a single locus (or very few loci) to polygenic adaptation at numerous sites. Best known are the models for single loci. Clearly, very strong positive directional selection at a single locus may explain fast adaptation, such as in the case of the peppered moth, as proposed by Haldane [9]. Haldane’s deterministic model was extended in several directions to make it suitable for data analysis. The extension proposed by Maynard Smith and Haigh [10] in 1974, 50 years after Haldane’s 1924 paper, for studying genetic hitchhiking is most valuable.

In more recent years, positive selection was combined with other factors characterizing natural populations, in particular, genetic drift, to investigate the hitchhiking effect in natural populations [11]. Purifying selection (in particular, background selection) was integrated into the analysis of genetic data [12]. The demographic history, such as changes of the population size, was also considered, and the population structure was included to describe typical phenomena, such as local adaptation [13,14,15,16]. Together, this work led to the development of numerous methods for finding evidence of adaptive evolution in genetic data [17,18,19,20].

On the other hand, polygenic adaptation caused by many weakly selected loci of small effects is not nearly as well studied as strong positive selection, leading to selective sweeps [21]. The interest of population geneticists in this type of selection was only very recently evoked by Pritchard and colleagues [22,23]. These authors suggested that allele frequencies may change by small amounts when a large number of genetic loci of small effects govern a quantitative trait, but whether such polygenic selection can explain rapid adaptation is unclear. 

The quantitative genetic analysis of models of polygenic adaptation has a relatively long history [24,25,26]. However, it is not a goal of this paper to review the history of this field. Instead, we present an overview of the recent literature on polygenic adaptation that is based on population genetic theory. De Vladar and Barton [27] analyzed a deterministic model originally proposed by Wright [28]. This model predicts that rapid adaptation may occur either through strong positive selection at a few loci (when the effect sizes of the alleles at these loci are large, relative to a scaled mutation rate), through weak selection at many loci with small effect sizes or through a combination of these two extreme modes [29,30,31]. However, all these studies assumed infinitely large population sizes. This led to problems in defining the values of allele frequencies at the time of environmental change, as the equilibrium allele frequencies of the deterministic model may not agree with the frequencies typically observed in genome-wide association studies (GWAS).

The studies of the past few years [32,33,34,35,36,37] incorporate genetic drift (due to finite population size) into their polygenic models. Simons et al. [34] proposed a model of selection that simultaneously acts on multiple traits (pleiotropy), which is frequently observed by GWAS in humans [38]. Stetter et al. [36] and Thornton [37] used extensive forward simulations to analyze a model (though with relatively few selected loci) that also includes neutral loci linked to selected ones. Stetter et al. also investigated the effect of different demographies and genetic architectures of the trait. In the model of Höllinger et al. [32], the loci controlling a trait are not explicitly given, but instead a genome-wide mutation rate is used as a proxy. Their main conclusions on the modes of rapid adaptation, however, are very similar to the predictions of Jain and Stephan [29,30] mentioned above.

In this paper, we review the theoretical work on rapid adaptation based on a model of a single quantitative trait that was analyzed by de Vladar and Barton [27], Jain and Stephan [29,30,31], John and Stephan [33], and Stephan and John [35]. This model covers the entire range of genetic architectures, from a few loci of major effects to the highly polygenic case, and has been studied for very large (infinite) populations and for populations of finite size.

## 2. Deterministic Model 

We assume that a trait is perfectly heritable and controlled additively (no dominance or epistasis) by *n* unlinked, diallelic loci in a very large population of diploids. If the phenotypic effect of the trait-increasing allele, also called “+” allele, at locus *i* is $\frac{\gamma_{i}\;}{2}$ and that of the “−” allele is $\frac{-\gamma_{i}\;}{2}$, the mean phenotype $c_{1}$, the genetic variance $c_{2}$ and the skewness $c_{3}$ are given by the following [30]:

(1) $$c_{1}=\sum_{i=1}^{n}\gamma_{i}\left(p_{i}-q_{i}\right)=\sum_{i=1}^{n}\gamma_{i}\left(2p_{i}-1\right)\;,\;$$

(2) $$c_{2}=2\sum_{i=1}^{n}\gamma_{i}^{2}p_{i}q_{i\;\;},\;\mathrm{and}$$

(3) $$c_{3}=2\sum_{i=1}^{n}\gamma_{i}^{3}p_{i}q_{i\;\;}\left(q_{i}-p_{i}\right)\;,$$

where $p_{i}$ is the frequency of the “+” allele at locus *i* and $q_{i}=1-p_{i}$ is that of the “−” allele. The effect sizes are assumed to be exponentially distributed with mean $\bar{\gamma }$. Furthermore, the fitness w of an individual with trait value *z* has a Gaussian shape centered about the fitness optimum, which in equilibrium is called $z_{0}$. Thus,

(4) $$w\left(z\right)=e^{-\frac{s}{2}{\left(z-z_{0}\right)}^{2}},$$

where *s* quantifies the strength of selection on the trait, such that 1/*s* is much larger than the phenotypic variance [34]. We also assume $0<z_{0}$, and require that $z_{0}<n\bar{\gamma }.$ The latter condition ensures that—after a perturbation—the population mean converges to a stationary state close to the fitness optimum [29]. In a randomly mating population, the change in the frequency of the trait-increasing allele at the *i*th locus due to selection and mutation is then given by the following:

(5) $$\frac{dp_{i}}{dt}=-s\gamma_{i}p_{i}q_{i}\Delta c_{1}-\frac{s\gamma_{i}^{2}}{2}p_{i}q_{i}\left(q_{i}-p_{i}\right)-\mu \left(p_{i}-q_{i}\right)\;,\;\;i=1,\ldots ,\;n,\;$$

where $\Delta c_{1}=c_{1}-z_{0}$ is the deviation of the mean phenotype from the fitness optimum. The first term on the right-hand side of Equation (5) describes positive directional selection toward the phenotypic optimum, the second term represents stabilizing selection in the neighborhood of the optimum [28], and the last term accounts for symmetric mutation between the “+” and “−” alleles [39,40]. 

De Vladar and Barton [27] analyzed the equilibrium properties of this model. They found that the alleles may be classified into those with effects smaller than a threshold value $\hat{\gamma }$ $=2\sqrt{\frac{2\mu }{s}\;}\;$(minor alleles) and those with larger sizes (major alleles). If the phenotypic mean coincides with the fitness optimum, the equilibrium frequency of the minor alleles is $\frac{1}{2}$, whereas the large-effect alleles are in a balance between mutation and selection near zero or one. If the phenotypic mean slightly deviates from the optimum, the equilibrium frequencies of the minor alleles are intermediate around one half (see Figure 2A of [27]).

This model is different from the so-called infinitesimal model, which is also widely used [26,41]. In the latter model, a large number of loci with very small effects control a quantitative trait such that the genetic variance in equilibrium does not depend on the number of loci. In contrast, in our model, we assume that the effect sizes do not change with the number of loci, but the variance may change substantially.

## 3. Two Extreme Scenarios of Rapid Adaptation

To describe the dynamics of rapid adaptation based on our deterministic model, we assume that the population is in equilibrium with no deviation from the phenotypic optimum located at $z_{0}$ [27]. This assumption is a useful approximation; it is elaborated in the next section. Then, after the optimum is suddenly shifted to another value $z_{f}$, the allele frequencies evolve in time toward the new stationary state. We assume that $z_{0}<$ $z_{f}<n\bar{\gamma }$. The latter assumption ensures that the trait mean approaches a value close to the new optimum. Thus, in contrast to Fisher’s [42] geometric model, it does not take several bouts of adaptation to reach the optimum. 

In the short-term phase, i.e., the time until the phenotypic mean reaches a value close to the new optimum, ODEs (5) can be approximated by the following [29,30]:

(6) $${\dot{p}}_{i}=S_{i}p_{i}q_{i},$$

where $S_{i}=-s\gamma_{i}\;\Delta c_{1}\left(t\right)$ and $\Delta c_{1}=c_{1}-z_{f}$. This approximation is based on the insight that in the initial phase when the population is far from the new fitness optimum, the dynamics described by ODEs (5) are dominated by directional selection rather than stabilizing selection and mutation.

Equation (6) is formally identical to the classical model of directional selection. However, the selection term depends on the distance from the new phenotypic optimum. Furthermore, because the mean deviation $\Delta c_{1}\left(t\right)$ contains a sum over all allele frequencies, Equation (6) is coupled. However, Jain and Stephan [30] found approximate solutions for both extremes: (i) the case that most (if not all) loci have small effects and (ii) the case that most (all) effects are large. To obtain analytical results, we usually assume in case (i) that $\gamma_{i}$ < $\hat{\gamma }$, where $\hat{\gamma }$ $=2\sqrt{\frac{2\mu }{s}\;}$ [13], and in case (ii) $\gamma_{i}$ > $\hat{\gamma }$.

First, we consider how the trait mean converges toward the new optimum in these two cases. In case (i), when most effects are small, the new optimum is approached exponentially at rate $sn{\bar{\gamma }}^{2}$, where, in this case, n is the number of minor loci. In case (ii), the rate is proportional to $sz_{f}\bar{\gamma }$. Thus, when most effects are small, the rate depends linearly on the number of loci involved. Hence, if n is large, adaptation may be rapid. In other words, in case (i), rapid adaptation is due to the fact that the equilibrium variance is large because the initial allele frequencies are intermediate (see above). Indeed, the equilibrium genetic variance before the phenotypic optimum is shifted to another value is given by $n{\bar{\gamma }}^{2}$ [27,29]. In contrast, in case (ii) in which most effects are large, the equilibrium genetic variance is small, as the initial allele frequencies are in a mutation—selection balance close to the boundaries 0 and 1. In this case, only a few large-effect loci are important in the short-term phase, and the rate of adaptation is determined by their large phenotypic effects rather than the number of loci (see Equation (30) in [30]).

Second, our analysis also revealed large differences in the dynamics of allele frequencies for large- versus small-effect loci [29,30]. In case (i), the time dependence of the allele frequencies for minor loci can be obtained analytically using Equation (6) and the formula for the exponential approach to the optimum [26,43]. These shifts in allele frequency are small to moderate, as verbally predicted by [22,23]. In contrast, when most effects are large, rapid fixations leading to selective sweeps and also other qualitatively different features of allele frequency trajectories at major loci may be observed (see Figure 3 in [30]). 

Equation (6) also allows us to predict the minimum effect size required for rapid fixation (selective sweep) to occur at a major locus. When most effects are large, we find that the allele frequency at a locus with an effect size larger than the mean effect may undergo a large shift (see Equation (41) in [30]). Therefore, selective sweeps are expected to occur at several major loci when many large-effect loci determine a trait. On the other hand, when most effects are small, we find that an effect size larger than the initial variance is required for a large change in the allele frequency (see Equation (38) in [30]); however, for exponentially distributed effects, the probability of such events is very small for a large number of loci. Therefore, selective sweeps are unlikely when a phenotypic trait is controlled by many loci of small effects.

Finally, when most effects are large such that rapid fixations occur, we often find large allele frequency shifts that resemble fixation processes. However, they are very slow and thus not observed within the short-term phase in which classical sweeps occur. Such an example can be found in Figure 3 in [30]. In this example, an allele increases from a low to a very high frequency on a timescale that is about three orders of magnitude larger than the short-term phase. Such an allele does not cause features that are known for selective sweeps (e.g., a strong reduction of neutral variation around the selected locus). Therefore, this allele would probably remain undetected by the available methods used to identify selective sweeps [44]. Another often observed pattern is that trajectories, which initially increase like alleles going to fixation, level off or even decrease with time (see the example in Figure 3 in [30]). This is typical for effect sizes that are large but not among the largest ones. It suggests that lower-ranked alleles are out-competed by the largest-effect ones.

The aforementioned scenarios leading to fast adaptation are extremes on a scale, which—in reality—comprises combinations of processes that range from fixations due to strong selection (causing selective sweeps) at a few loci to weak selection at many loci (polygenic adaptation with subtle allele frequency changes). In contrast to case (ii), in the case of polygenic adaptation due to weak selection at many loci, genetic drift is expected to play an important role. This subject is treated in the following section.

## 4. The Role of Genetic Drift in Rapid Adaptation

In this section, we consider biologically more realistic conditions for adaptation than above by integrating genetic drift into our deterministic model. We show that in the stochastic equilibrium between genetic drift, mutation and selection, the population mean is fluctuating around a state close to the fitness optimum $z_{0}$ (but not identical to $z_{0}$). Then, after the optimum is shifted suddenly to a new value $z_{f}$, the population will adapt and evolve toward a new stationary state. To understand the role of genetic drift in both the equilibrium phase and the adaptive phase, we essentially follow the treatments of John and Stephan [33] and Stephan and John [35], and assume that most of the loci have small effects such that $\gamma_{i}$ < $\hat{\gamma }$, where $\hat{\gamma }$ $=2\sqrt{\frac{2\mu }{s}\;}$ [13]. The (diploid) population size is N, which is assumed to be constant.

Since we learned that in the deterministic case, the trait mean may change much faster after a perturbation than the allele frequencies [29], we express Equation (5) as follows:

(7) $$\frac{d\Delta c_{1}}{dt}=-\frac{s}{2}c_{3}-sc_{2}\Delta c_{1}-2\mu c_{1}$$

and

(8) $$\frac{dp_{i}}{dt}=-s\gamma_{i}p_{i}q_{i}\Delta c_{1}-\frac{s\gamma_{i}^{2}}{2}p_{i}q_{i}\left(q_{i}-p_{i}\right)+\mu (q_{i}-p_{i}).$$

Equation (7) is derived by summing over Equation (5) and using the definitions of the cumulants (Equation (1) to Equation (3)). Since in the case of rapid evolution $Ɗc_{1}$ is a fast variable on the timescale of the allele frequencies $p_{i}$ (see above), we obtain in the quasi-equilibrium state $\Delta {\tilde{c}}_{1}$ by equating the left-hand side of Equation (7) to zero [45] and obtain, approximately, the following:

(9) $$\Delta {\tilde{c}}_{1}\approx -\frac{\frac{s}{2}{\tilde{c}}_{3}+2\mu z_{0}}{s{\tilde{c}}_{2}+2\mu }\;,$$

where tilde indicates the quasi-equilibria of the cumulants involved. While the variance is relatively constant [29], the skewness varies with time but may be very small because the effect sizes are small in the polygenic case (Equation (3)). Equation (9) predicts that in equilibrium, the trait mean is not identical to, but very close to, the fitness optimum, because in this model, the allele frequencies of Equation (5) approach stable equilibrium states that are incompatible with the fitness optimum (see Figure 2A and Appendix B in [27]).

Using Equation (9), the expected change of the allele frequency $p_{i}$ may be approximated as follows:

(10) $$E\left\{\Delta p_{i}\right\}\approx -s\gamma_{i}p_{i}q_{i}\Delta {\tilde{c}}_{1}-\frac{s\gamma_{i}^{2}}{2}p_{i}q_{i}\left(q_{i}-p_{i}\right)+\mu (q_{i}-p_{i}),$$

where $\Delta {\tilde{c}}_{1}$ is assumed to be constant. Furthermore, the variance of the change in $p_{i}$ is as follows:

(11) $$Var\left\{\Delta p_{i}\right\}\approx \frac{p_{i}q_{i}}{2N}.$$

Using diffusion theory [46], we obtain the equilibrium frequency distribution of the trait-increasing allele $p_{i}$ at locus *i* as follows:

(12) $$f\left(p_{i}\right)\approx C_{i}p_{i}^{2\beta -1}q_{i}^{2\beta -1}exp\left(-2\alpha \gamma_{i}\Delta {\tilde{c}}_{1}p_{i}-\alpha \gamma_{i}^{2}p_{i}q_{i}\right),$$

where $C_{i}$ is the normalization constant, α=2Ns, and β=2Nμ is the scaled mutation rate. $C_{i}$ is approximately given by the following [33]:

(13) $$C_{i}{}^{-1}\approx B(2\beta ,2\beta )\;\left[1-\alpha \gamma_{i}\Delta {\tilde{c}}_{1}-\alpha \gamma_{i}^{2}\frac{\beta }{4\beta +1}\right],$$

where *B* denotes the beta function.

**Figure 1:**
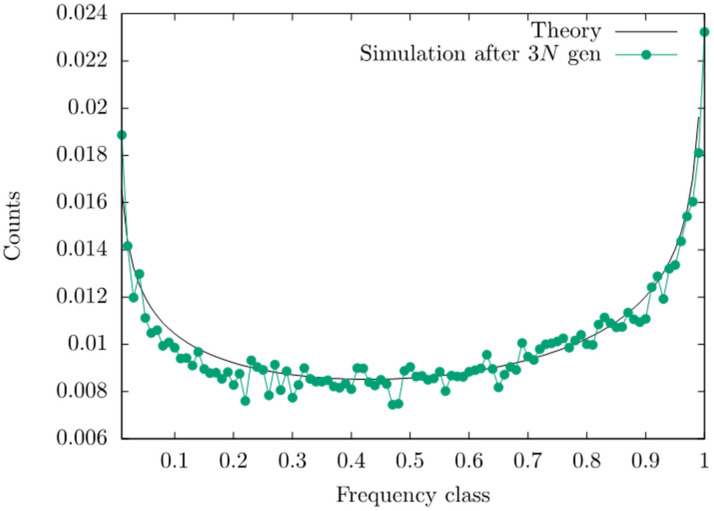
Equilibrium distribution of allele frequencies for a locus with γ=0.0107. Simulations are described in [26] and were run for 3N generations. The solid curve is predicted by Equations (12) and (13). The parameter values are *s* = 0.1, *N* = $2\times 10^{4}$, *n* = 200, μ = 10^−5^, $\bar{\gamma }=0.01$, $z_{0}$ = 0.2, and $\Delta {\tilde{c}}_{1}=-0.0022$.

We compare Equations (12) and (13) with simulations, using the following set of parameter values: *s* = 0.1, *N* = $2\times 10^{4}$, *n* = 200, μ = 10^−5^ and $\bar{\gamma }=0.01$. These values are from the literature on polygenic adaptation in humans [8,34]; the value for the optimum $z_{0}$ was chosen such that $z_{0}$ = 0.2 < $n\bar{\gamma }$. We find an excellent agreement of the theoretical predictions with the simulation results (Figure 1). This fit is better than the original one in Figure 2 in [33] because the simulations were run for a longer time. 

An important difference to the deterministic model is that in our stochastic analysis, the equilibrium is given as a probability distribution of allele frequencies (Equation (12)), not as a single value (as in the deterministic model). For very large populations, this distribution is bell-shaped if β>0.5. However, for our set of biologically realistic parameter values, we find a U-shaped distribution (Figure 1). The reason is that in equilibrium, the genetic variance for exponentially distributed effect sizes with mean $\bar{\gamma \;}$ is approximately $\frac{4\beta }{4\beta +1}n{\bar{\gamma }}^{2}$ rather than $n{\bar{\gamma }}^{2}$ [33].

To model the adaptation to the new fitness optimum, we consider a population that is in equilibrium when the fitness optimum $z_{0}$ is suddenly shifted to a new value $z_{f}>z_{0}$, where $z_{f}<n\bar{\gamma }$. As explained in the previous section, since the trait mean responds rapidly to the shift of the fitness optimum, it may be approximated by an exponential process with a rate that is proportional to the equilibrium genetic variance at the time of the environmental shift of the optimum. 

Next, we investigate the effect of drift on the genetic variance in the adaptive phase. We describe the stochastic changes of the frequencies of the trait-increasing alleles $p_{i}$, using a diffusion approximation (similar to the approach of [47]). The differential operator ${ℒ}_{i}$ of the Kolmogorov backward equation is the following [47] :

(14) $${ℒ}_{i}=\left(-s\gamma_{i}p_{i}\left(1-p_{i}\right)\Delta c_{1}\left(t\right)\right)\frac{\partial }{\partial p_{i}}+\frac{p_{i}\left(1-p_{i}\right)}{4N}\frac{\partial^{2}}{\partial p_{i}^{2}}.$$

As in Equation (6), the selection term of this operator includes only the effect of directional selection. Using Equation (14), we obtain ODEs for the lowest-order moments of the allele frequencies as follows [46]:

(15) $$\frac{d}{dt}E\left\{p_{i}\right\}=-s\gamma_{i}E\left\{p_{i}\left(1-p_{i}\right)\right\}\Delta c_{1}\left(t\right),$$

(16) $$\;\frac{d}{dt}E\left\{p_{i}^{2}\right\}=-2s\gamma_{i}E\left\{p_{i}^{2}\left(1-p_{i}\right)\right\}\Delta c_{1}\left(t\right)+\frac{1}{2N}E\left\{p_{i}\left(1-p_{i}\right)\right\},$$

and combining these two ODEs yields the following:

(17) $$\frac{d}{dt}E\left\{p_{i}\left(1-p_{i}\right)\right\}=-s\gamma_{i}E\left\{p_{i}\left(1-p_{i}\right)\right\}\Delta c_{1}\left(t\right)+2s\gamma_{i}E\left\{p_{i}^{2}\left(1-p_{i}\right)\right\}\Delta c_{1}\left(t\right)\;-\frac{1}{2N}E\left\{p_{i}\left(1-p_{i}\right)\right\}.$$

Equation (17), in combination with Equation (2), suggests that the contribution of individual loci to genetic variance decreases with increasing genetic drift. As the last term on the right-hand side of Equation (17) is negative and proportional to $\frac{1}{2N}$, a smaller population size (and, thus, stronger drift) reduces the contribution of a locus to genetic variance. In other words, drift reduces genetic variance during the adaptive phase. 

Finally, we use simulations to explore the effect of genetic drift on the dynamics of allele frequencies in the adaptive phase [35]. Of particular interest are the frequency shifts $\delta p_{i}$ of the alleles at locus i during the short-term phase. Since we assume that $z_{f}>z_{0}$ and thus $\Delta c_{1}\left(0\right)<0$, the allele frequencies $p_{i}\left(t\right)$ are expected to increase with time (Equation (15)). In the deterministic case, the allele frequency shifts at the end of the short-term phase for sufficiently small effect sizes are approximately the following [33]:

(18) $$\delta p_{i}\approx -\gamma_{i}p_{i}\left(0\right)q_{i}\left(0\right)\Delta c_{1}\left(0\right)\frac{1-e^{-1}}{c_{2}\left(0\right)}.$$

Equation (18) suggests that the allele frequency shift at a locus depends strongly on the compound parameter $\gamma_{i}p_{i}\left(0\right)q_{i}\left(0\right)$. This parameter increases with the effect size and is greatest for initial frequencies that are intermediate. Furthermore, Equation (18) predicts that after an environmental change, the allele frequencies shift coherently into the same direction. This is an important property of polygenic selection, as it may help in detecting this type of selection (see discussion section), although the frequency shifts at individual loci are in general small. 

Including genetic drift, however, leads to a more complex scenario of polygenic adaptation. We find a good agreement between the theory and simulation for the deviation $\Delta c_{1}$ of the population mean from the fitness optimum within the short-term phase [35]. For the allele frequencies, however, we observe a reasonable agreement of the deterministic prediction of Equation (18) and simulation only when the effect sizes are sufficiently large and allele frequencies at the time of the environmental shift are intermediate. This is a consequence of the U-shaped equilibrium distribution of allele frequencies (see Figure 1). As revealed by Equations (15) and (17), the reason is that genetic drift slows down the increase in the allele frequencies and, thus, reduces the expected differences between the allele frequencies at the end of the short-term phase and those at the time of the optimum shift. Thus, trait-increasing alleles with intermediately high initial equilibrium frequencies contribute positively to changes of the trait mean (i.e., are aligned with the direction of the optimum shift), whereas alleles with low or high initial frequencies may not stay aligned with the optimum shift. Rapid polygenic adaptation in small natural or experimental populations is, therefore, very hard to detect.

## 5. Discussion

We analyzed a deterministic model to describe the short-term response of a quantitative trait after an environmental change, leading to a sudden shift of the fitness optimum. We provided approximate formulas for the timescales over which the trait mean approaches the new optimum. When the effect sizes are small, subtle to moderate allele frequency shifts occur within the phase of fast adaptation. In contrast, dramatic frequency changes (including fixations leading to selective sweeps) may be observed for large effect sizes. 

We also integrated random genetic drift into our model to analyze polygenic adaptation, i.e., the case of small effect sizes and weak selection at many loci. We investigated the equilibrium distribution of allele frequencies (before the environmental shift), based on diffusion theory. For realistic values of population sizes and mutation rates, this distribution is U-shaped. We also studied the adaptive phase. We found that—as in the deterministic model—the trait mean approaches the new optimum exponentially at a rate proportional to the equilibrium genetic variance. This result agrees with Lande’s [26] prediction of a selection response based on the infinitesimal model. However, the dynamics of allele frequencies may differ significantly from those of the deterministic model, due to their U-shaped equilibrium distribution. Only alleles with intermediately large equilibrium frequencies contribute positively to changes in the trait mean (i.e., are aligned with the optimum shift). In contrast, alleles with very low or high frequencies are subject to stronger drift and, thus, may not stay aligned with the direction of the optimum shift.

The model we analyzed is simple in that the allelic effects on traits are assumed to be additive (no dominance or epistasis). Our basic assumption, however, fits well with the notion that many quantitative traits are polygenic and that mutations of small effect at a locus tend to act additively. These findings are consistent with observations of GWAS in humans in which the variants identified rarely have large effects (reviewed in [38]).

Our findings have some important implications for the detection of positive selection associated with quantitative traits. Our results suggest that the identification of polygenic selection in the genome may be hindered by the effects of genetic drift. All methods that were proposed for identifying polygenic selection are based on combining signals of changes in allele frequency across many loci that control a given trait and testing whether these changes tend to affect the trait in a certain direction [38]. The detection of polygenic selection is facilitated when the allele frequencies shift in the same direction after an environmental change [31], as predicted by the deterministic model (see Equation (18)). However, in a finite population experiencing drift, the situation is more complicated, as only for sufficiently large values of the parameter $\gamma_{i}p_{i}\left(0\right)q_{i}\left(0\right)$ the frequency shifts of the trait-increasing alleles occur in the same direction. 

Despite these difficulties, there are several reports on the detection of rapid polygenic adaptation in the literature. The best-studied trait is human height [8,48]. Turchin et al. [8] compared two classes of populations that separated from each other in the past ca. 100 generations: southern and northern European populations. In their analysis, they assumed that in the southern populations, only genetic drift was present, while in the northern ones, drift and polygenic selection were operating. Selection was modeled as in Equation (6). However, the deviation of the trait mean from the optimum was not taken into account. Based on data from GWAS, they showed that trait-increasing alleles (“+” alleles) were significantly more frequent in northern populations (Sweden and the U.K.) than in southern ones (Italy, Spain and Portugal). They concluded, based on likelihood ratio tests, that the observed frequency differences are better explained by the model that comprises both drift and selection rather than drift alone. A similar test was proposed by Berg and Coop [18]; it also relies on frequency differencies of trait-increasing alleles between extant populations.

Another approach to detect signatures of polygenic adaptation was introduced by Field et al. [48]. It is based on the idea that a terminal branch of a genealogy is shorter for a beneficial allele than for a detrimental one. They applied their method to a single population from Britain and found that the frequency of height-increasing alleles increased significantly in the past 80 generations (ca. 2000 years). Recently a similar yet more general method was proposed by Edge and Coop [49]. It relies on explicit inferences of the genealogies of variants associated with a trait rather than summary statistics of tip-branch lengths.

Classical sweeps, on the other hand, that occur when effect sizes are large (see above) can be identified by several methods developed in the past 20 years (reviewed in [44]). In natural populations, selective sweeps at quantitative trait loci (QTL) may be relatively rare [43,50,51]. In contrast, in domesticated populations of, for example, pigs, chicken and cattle, many examples have been detected in which selective sweeps overlap with known QTL [52,53,54]. These observations may be attributed to the action of artificial selection during domestication, which causes larger optimum shifts and, thus, more opportunities for sweeps than selection in natural populations (see Equation (41) in [30]).

Finally, we discussed the effect of population size bottlenecks on polygenic adaptation. In [35], we described simulations of a bottleneck, which mimics part of recent human history: an effective population size of $2\times 10^{4}$ decreased after the out-of-Africa migration to 3000 individuals and recovered after 5000 generations back to the original size [55]. During the bottleneck, the deviation of the trait mean from the optimum increased by about 41%, while genetic variance decreased by about the same amount (43%). Both observations are roughly in agreement with Equation (9), and the decay of genetic variance follows, approximately, Equation (17).

This example suggests that genetic drift associated with severe bottlenecks may prevent quantitative traits from appproaching their fitness optimum very closely. Therefore, observing rapid polygenic adaptation, e.g., in experimental populations, is expected to be very difficult, unless their source populations are large. In summary, our analysis of genetic drift in rapid adaptation may indicate that in some of the well-known examples of rapid adaptation (such as in experimental populations of anoles, mentioned in the introduction [6]), polygenic selection was probably not a major driving force, unless it acted in combination with strong positive directional selection at a few loci.

## Data Availability

The data (for Figure 1) are contained within the article.

## References

[1] Reznick D.N. (2009). The Origin Then and Now: An Interpretive Guide to the Origin of Species.

[2] Vignieri S.N., Larson J.G., Hoekstra H.E. (2010). The selective advantage of crypsis in mice. Evolution.

[3] Cook L.M., Grant B.S., Saccheri I.J., Mallet J. (2012). Selective bird predation on the peppered moth: The last experiment of Michael Majerus. Biol. Lett..

[4] Ffrench-Constant R.H., Bogwitz M., Daborne P., Yen J. (2002). A single P450 allele associated with insecticide resistance in *Drosophila*. Science.

[5] Grant P.R., Grant B.R. (2008). How and Why Species Multiply: The Radiation of Darwin’s Finches.

[6] Losos J.B. (2009). Lizards in an Evolutionary Tree: Ecology and Adaptive Radiation of Anoles.

[7] van’t Hof A.E., Edmonds N., Dalikova M., Marec F., Saccheri I.J. (2011). Industrial melanism in British peppered moths has a singular and recent mutational origin. Science.

[8] Turchin M.C., Chiang C.W.K., Palmer C.D., Sankararaman S., Reich D. (2012). Genetic Investigation of ANthropometric Traits (GIANT) Consortium; Hirschhorn, J.N. Evidence of widespread selection on standing variation in Europe at height-associated SNPs. Nat. Genet..

[9] Haldane J.B.S. (1924). A methematical theory of natural and artificial selection. Part, I. Trans. Camb. Philos. Soc..

[10] Maynard Smith J., Haigh J. (1974). Hitchhiking effect of a favourable gene. Genet. Res..

[11] Kaplan N.L., Hudson R.R., Langley C.H. (1989). The ‘hitchhiking effect’ revisited. Genetics.

[12] Charlesworth B., Morgan M.T., Charlesworth D. (1993). The effect of deleterious mutations on neutral molecular variation. Genetics.

[13] Catalan A., Glaser-Schmitt A., Argyridou E., Duchen P., Parsch J. (2016). An indel polymorphism in the *MtnA* 3′ untranslated region is associated with gene expression variation and local adaptation in *Drosophila melanogaster*. PLoS Genet..

[14] Lange B.W., Langley C.H., Stephan W. (1990). Molecular evolution of Drosophila metallothionein genes. Genetics.

[15] Pfeifer S.P., Laurent S., Sousa V., Linnen C.R., Foll M., Excoffier L., Hoekstra H.E., Jensen J.D. (2018). The evolutionary history of Nebraska deer mice: Local adaptation in the face of strong gene flow. Mol. Biol. Evol..

[16] Slatkin M., Wiehe T. (1998). Genetic hitch-hiking in a subdivided population. Genet. Res..

[17] Akey J.M., Eberle M.A., Rieder M.J., Carlson C.S., Shriver M.D., Nickerson D.A., Kruglyak L. (2004). Population history and natural selection shape patterns of genetic variation in 132 genes. PLoS Biol..

[18] Berg J.J., Coop G. (2014). A population genetic signal of polygenic adaptation. PLoS Genet..

[19] Kim Y., Stephan W. (2002). Detecting a local signature of genetic hitchhiking along a recombining chromosome. Genetics.

[20] Nielsen R., Williamson S., Kim Y., Hubisz M.J., Clark A.G., Bustamante C. (2005). Genomic scans for selective sweeps using SNP data. Genome Res..

[21] Stephan W. (2019). Selective sweeps. Genetics.

[22] Pritchard J.K., Di Rienzo A. (2010). Adaptation–not by sweeps alone. Nat. Rev. Genet..

[23] Pritchard J.K., Pickrell J.K., Coop G. (2010). The genetics of human adaptation: Hard sweeps, soft sweeps, and polygenic adaptation. Curr. Biol..

[24] Bürger R. (2000). The Mathematical Theory of Selection, Recombination, and Mutation.

[25] Bürger R., Lynch M. (1995). Evolution and extinction in a changing environment: A quantitative-genetic analysis. Evolution.

[26] Lande R. (1976). Natural selection and random genetic drift in phenotypic evolution. Evolution.

[27] De Vladar H.P., Barton N. (2014). Stability and response of polygenic traits to stabilizing selection and mutation. Genetics.

[28] Wright S. (1935). Evolution in populations in approximate equilibrium. J. Genet..

[29] Jain K., Stephan W. (2015). Response of polygenic traits under stabilizing selection and mutation when loci have unequal effects. G3-Genes Genomes Genet..

[30] Jain K., Stephan W. (2017). Rapid adaptation of a polygenic trait after a sudden environmental shift. Genetics.

[31] Jain K., Stephan W. (2017). Modes of rapid polygenic adaptation. Mol. Biol. Evol..

[32] Höllinger I., Pennings P., Hermisson J. (2019). Polygenic adaptation: From sweeps to subtle frequency shifts. PLoS Genet..

[33] John S., Stephan W. (2020). Important role of genetic drift in rapid polygenic adaptation. Ecol. Evol..

[34] Simons Y.B., Bullaughey K., Hudson R.R., Sella G. (2018). A population genetic interpretation of GWAS findings for human quantitative traits. PLoS Biol..

[35] Stephan W., John S. (2020). Polygenic adaptation in a population of finite size. Entropy.

[36] Stetter M.G., Thornton K., Ross-Ibarra J. (2018). Genetic architecture and selective sweeps after polygenic adaptation to distant optima. PLoS Genet..

[37] Thornton K.R. (2019). Polygenic adaptation to an environmental shift: Temporal dynamics of variation under Gaussian stabilizing selection and additive effects on a single trait. Genetics.

[38] Sella G., Barton N.H. (2019). Thinking about the evolution of complex traits in the era of genome-wide association studies. Annu. Rev. Genom. Hum. Genet..

[39] Barton N.H. (1986). The maintenance of polygenic variation through a balance between mutation and stabilizing selection. Genet. Res..

[40] Bulmer M.G. (1972). The genetic variability of polygenic characters under optimizing selection, mutation and drift. Genet. Res..

[41] Barton N.H., Etheridge A.M., Veber A. (2017). The inifinitesimal model: Definition, derivation, and implications. Theor. Popul. Biol..

[42] Fisher R.A. (1930). The Genetical Theory of Natural Selection.

[43] Chevin L.-M., Hospital F. (2008). Selective sweep at a quantitative trait locus in the presence of background genetic variation. Genetics.

[44] Stephan W. (2016). Signatures of positive selection: From selective sweeps at individual loci to subtle allele frequency changes in polygenic adaptation. Mol. Ecol..

[45] Gardiner C.W. (1990). Handbook of Stochastic Methods.

[46] Ewens W.J. (2004). Mathematical Population Genetics. I. Theoretical Introduction.

[47] Stephan W., Wiehe T.H.E., Lenz M.W. (1992). The effect of strongly selected substitutions on neutral polymorphism: Analytical results based on diffusion theory. Theor. Popul. Biol..

[48] Field Y., Boyle E.A., Telis N., Gao Z., Gaulton K.J., Golan D., Yengo L., Rocheleau G., Froguel P., McCarthy M.I. (2016). Detection of human adaptation during the past 2000 years. Science.

[49] Edge M.D., Coop G. (2019). Reconstructing the history of polygenic scores using coalescent trees. Genetics.

[50] Pavlidis P., Metzler D., Stephan W. (2012). Selective sweeps in multi-locus models of quantitative traits. Genetics.

[51] Wollstein A., Stephan W. (2014). Adaptive fixation in two-locus models of stabilizing selection and genetic drift. Genetics.

[52] Axelson E., Ratnakumar A., Arendt M.L., Maqbool K., Webster M.T., Perloski M., Liberg O., Arnemo J.M., Hedhammar Å., Lindblad-Toh K. (2013). The genomic signature of dog domestication reveals adaptation to a starch-rich diet. Nature.

[53] Qanbari S., Pausch H., Jansen S., Somel M., Strom T.M., Fries R., Nielsen R., Simianer H. (2014). Classic selective sweeps revealed by massive sequencing in cattle. PLoS Genet..

[54] Rubin C.J., Megens H.J., Barrio A.M., Maqbool K., Sayyab S., Schwochow D., Wang C., Carlborg Ö., Jern P., Jørgensen C.B. (2012). Strong signatures of selection in the domestic pig genome. Proc. Natl. Acad. Sci. USA.

[55] Schiffels S., Durbin R. (2014). Inferring human population size and separation history from multiple genome sequences. Nat. Genet..

